# Identification of Differentially Expressed Genes and Molecular Pathways Involved in Primary Biliary Cholangitis Using RNA-Seq

**DOI:** 10.3390/genes17010010

**Published:** 2025-12-22

**Authors:** Min Yang, Xiaoyun Shen, Haitao Fu, Jie Lu, Fengying Li

**Affiliations:** 1 Department of Clinical Laboratory, Sir Run Run Shaw Hospital, School of Medicine, Zhejiang University, Hangzhou 310016, China; yang_min@zju.edu.cn (M.Y.); jielu1027@126.com (J.L.); 2 Key Laboratory of Precision Medicine in Diagnosis and Monitoring Research of Zhejiang Province, Hangzhou 310016, China; 3 Key Laboratory of Endoscopic Technology Research, Sir Run Run Shaw Hospital, Zhejiang University School of Medicine, Hangzhou 310016, China; xiaoyun_shen@zju.edu.cn; 4 Department of Laboratory and Blood Transfusion, The 904th Hospital of PLA Joint Logistics Support Force, Wuxi 214007, China; fuhaotao@hotmail.com

**Keywords:** PBC, RNA-Seq, STX17-DT, proliferation, apoptosis

## Abstract

**Objective**: This study aims to investigate the functional role of lncRNA STX17-DT, which was previously found to be upregulated in peripheral blood mononuclear cells (PBMCs) of PBC patients, by examining its impact on gene expression and cellular behavior in a human monocyte model. **Methods**: STX17-DT was overexpressed in THP-1 cells, which was assessed via plasmid transfection. Transcriptomic changes were analyzed by RNA sequencing, followed by comprehensive bioinformatics analyses including differential expression, functional enrichment, transcription factor network, and protein–protein interaction (PPI) analysis. Functional validation was performed using CCK-8 and TUNEL assays to assess proliferation and apoptosis, respectively. **Results**: Overexpression of STX17-DT led to 1973 differentially expressed genes (DEGs), with 1201 upregulated and 772 downregulated. Key upregulated genes included interferon-stimulated genes (e.g., *interferon induced protein 44 like* (*IFI44L*), *interferon induced protein 44* (*IFI44*), *guanylate binding protein 1*(*GBP1*)) and chemokines (*CCL4*, *CCL8*). Upregulated DEGs were significantly enriched in immune-related pathways such as NF-κB signaling, Toll-like receptor signaling, TNF signaling, and cytokine–cytokine receptor interaction. Downregulated genes were involved in metabolic and signaling pathways such as PI3K–Akt, cAMP, and butanoate metabolism. Transcription factor analysis revealed significant alterations in regulators like *Yes1 associated transcriptional regulator*(*YAP1*), *nuclear receptor subfamily 4 group A member 1*(*NR4A1*), and *MAF bZIP transcription factor B*(*MAFB*). PPI network analysis suggested *TNF*, *TLR4*, *TLR6*, and *STAT2* as central hubs. Functionally, STX17-DT overexpression enhanced THP-1 cell proliferation and significantly reduced apoptosis. **Conclusions**: STX17-DT promoted a pro-inflammatory transcriptomic profile and enhanced monocyte survival in our study, suggesting a potential role in PBC immunopathology. It may represent a potential biomarker and therapeutic target, particularly for patients with advanced disease or suboptimal response to ursodeoxycholic acid. Further studies in primary cells, animal models, and histological samples are warranted to validate its role in PBC pathogenesis.

## 1. Introduction

Primary biliary cholangitis (PBC), previously known as primary biliary cirrhosis, is a common autoimmune hepatic disease that mainly impacts middle-aged women [1]. This disease is characterized by a chronic humoral and cellular immune-driven destruction of the small bile ducts, resulting in serum specific anti-mitochondrial antibodies (AMAs) and histopathological evidence of degeneration and necrosis of intrahepatic biliary epithelial cells (BECs), surrounded by a dense infiltration of mononuclear cells [2]. The global epidemiology of PBC reveals an increasing healthcare burden, with recent meta-analyses reporting a worldwide prevalence of 18.1/100,000 and incidence of 1.8/100,000 person-years [3]. Approximately 40 percent of PBC patients present asymptomatically. Symptoms like fatigue, pruritus, jaundice, hepatomegaly, and splenomegaly typically emerge at more advanced histological stages when the prognosis is less favorable [4]. Previous studies have shown that nearly 40% early-diagnosed patients who promptly started ursodeoxycholic acid (UDCA) treatment remained non-progressive even 20 years after diagnosis [5]. This alarming statistic underscores persistent importance in early recognition, despite wider availability of diagnostic tools like MIT3-enhanced AMA testing and PBC-specific anti-nuclear antibodies (e.g., anti-gp210, sp100). Such delays inevitably compromise therapeutic efficacy; and several PBC patients have been diagnosed without manifesting positivity to the aforementioned auto-antibodies [6,7,8]. Despite its autoimmune features, PBC patients generally do not respond to immunosuppressive therapy, whereas they show a favorable response to UDCA, a first-line drug for cholestasis [9], and it also remains unclear why autoimmune responses preferably destroy small BECs in the liver of PBC patients regardless of the presence of AMAs.

Recent investigations have shed light on the intimate interactions between the fibrogenic hepatic stellate cell (HSC) and infiltrating immune cells, which fundamentally drive liver scarring [10]. Circulating innate immune cells like monocytes, serve as precursors to resident tissue macrophages and have been implicated in the pathogenesis of cholestasis. It has been well-established that peripheral blood mono-nuclear cells (PBMCs) can infiltrate into portal tracts and BECs [2], making them a valuable surrogate for investigating the systemic and liver-directed immune dysregulation in this disease [11,12]. This dysregulation is reflected in the spectrum of traditional PBC-specific autoantibodies. Beyond these, autoantibodies targeting nuclear body components like Sp140 have been identified and may be linked to aberrant antiviral immune responses, suggesting potential environmental triggers such as viral infections in the pathogenesis of PBC [13]. Therefore, studying molecular alterations within PBMCs can provide critical insights into the disease-specific immune features. In our previous study, we discovered that lncRNA STX17-DT was significantly upregulated in PBMCs of PBC patients compared to healthy control group [14]. THP-1 cells, a human leukemia monocyte cell line, differentiate into a macrophage-like phenotype when treated with phorbol 12-myristate 13-acetate(PMA) and serve as a commonly used and suitable in vitro model to investigate the pathogenic mechanisms involved in PBC [15,16]. In the current study, we overexpressed STX17-DT in THP1 cell line and used RNA-seq to identify genes that are differentially expressed. Bioinformatics analysis was performed to investigate the protein–protein interaction (PPI) and subnetwork modules, and to identify enriched signaling pathways to characterize the underlying pathogenic mechanisms.

## 2. Materials and Methods

### 2.1. Construction of the Recombinant Plasmid pcDNA-STX17-DT and Transfection of THP-1 Cells

The full-length STX17-DT sequence was retrieved from the NCBI database and cloned into the pcDNA3.1 vector to generate the pcDNA-STX17-DT expression plasmid. Both the STX17-DT amplicon and the pcDNA3.1 vector were digested with appropriate restriction enzymes and then ligated using DNA ligase to generate the recombinant plasmid. THP-1 cells (Homogene, Shanghai, China) were cultured in RPMI-1640 (Gibco, Thermo Fisher Scientific, Waltham, MA, USA) containing 10% FBS (Gibco, Thermo Fisher Scientific, USA) and 1% penicillin–streptomycin at 37 °C, 5% CO_2_. Then the pcDNA-STX17-DT recombinant plasmid was diluted in serum-free medium and mixed with the BeyoPEI™ transfection reagent (Beyotime, Shanghai, China) according to the manufacturer’s instructions. After incubation for 15–30 min at room temperature, the transfection complex was added to the cells. A control group of THP-1 cells was transfected with the empty pcDNA3.1 vector in parallel. Following transfection, cells were maintained under standard conditions for 48 h. STX17-DT overexpression was confirmed by RT-qPCR using the SYBR Green real-time PCR Master Mix and the following primers (sense: 5′-CCAATTCTACTACAGAAGGCAGAG-3′; anti-sense: 5′-GTCAGATTTAAGATTTGAGCCC-3′).

### 2.2. RNA Isolation and Library Preparation

Six cultured cell samples were analyzed, with three replicates each for STX17-DT overexpression group (named OE1, OE2, OE3) and the control group (CTRL1, CTRL2, CTRL3). Total RNA of each sample was extracted using TRizol (Thermo Fisher Scientific) reagent according to the manufacturer’s instructions. The purity and quantification of RNA were determined using a NanoDrop 2000 spectrophotometer (Thermo Fisher Scientific), and the integrity of RNA was assessed using an Agilent 2100 Bioanalyzer (Agilent Technologies, Santa Clara, CA, USA). The RNA-seq library was constructed using the VAHTS Universal V6 RNA-seq Library Prep Kit (Vazyme, Nanjing, China) according to the manufacturer’s instructions. The transcriptome sequencing and analysis were conducted by OE Biotech Co., Ltd. (Shanghai, China).

### 2.3. RNA Sequencing Process and Differentially Expressed Genes Analysis

The RNA libraries were sequenced by OE Biotech, Inc., Shanghai, China. We are grateful to them for assisting in sequencing and bioinformatics analysis. The library was sequenced on the Illumina Novaseq 6000 platform (Illumina, San Diego, CA, USA) to generate 150 bp paired-end reads. The raw reads in fastq format were processed using the fastp software (version 0.20.1) to remove low-quality reads, resulting in clean reads for subsequent data analysis. The expression of each protein-coding gene was identified by aligning the sequences to a database of known reference gene sequences and annotation files using sequence similarity alignment methods. The clean reads were then aligned to the reference genome using the HISAT2 software (version 2.1.0), and the expression of each gene in the samples was identified. FPKM (fragments per kilobase of transcript per million mapped reads) of each gene was calculated and the read counts for each gene were obtained by HTSeq-count (version 0.11.2). Principal Component Analysis (PCA) was performed on the gene counts using R software (version 4.2.0) to evaluate the biological replicates of the samples.

Differential expression analysis was performed using the DESeq2 software (version 1.22.2), and adjusted *p* value < 0.05 and |log_2_FoldChange| > 1 were set as the threshold for significantly differential expression gene (DEGs). Hierarchical cluster analysis of DEGs was performed using pheatmap (version 1.0.12) to demonstrate the expression pattern of genes in different groups and samples.

### 2.4. Bioinformatics Functional Analysis of DEGs

Based on the hypergeometric distribution, GO annotation, KEGG database and annotation, GO analysis and KEGG pathway enrichment analysis of DEGs were performed to screen the significant enriched term by clusterProfile (version 4.16.0), respectively. This platform was used to draw the column diagram, chord diagram and bubble diagram of the significant enrichment term. Gene Set Enrichment Analysis (GSEA) was performed using GSEA software (version 4.2.3) based on all detected genes and involved filtering gene sets, with default criteria being a minimum of 15 genes and a maximum of 500 genes per gene set, and these genes were ranked according to the degree of differential expression in two types of samples (OE and control group). Then, whether the predefined gene set was enriched at the top or bottom of the ranking list was tested.

### 2.5. Analysis of Transcription Factor and Target Gene Prediction

By comparing the distribution of transcription factors (TFs) among all genes and DEGs, transcription factors with significant proportional differences can be identified. These TFs can then be further analyzed for their potential association with the observed differences. Based on the list of relationships between transcription factors and their target genes, differentially expressed target genes corresponding to the differentially expressed TFs were extracted, and a statistical chart of target genes for differential transcription factor families was constructed.

### 2.6. Protein–Protein Interaction (PPI) Network and Module Analysis

A web-based tool, the Search Tool for the Retrieval of Interacting Genes (STRING) database, was utilized to generate a PPI network for up- and downregulated DEGs separately, with an interaction score > 0.4. The STRING output were analyzed by the Cytoscape software (v3.9.0) [17]. Cluster analyses of the PPI network for both up- and downregulated DEGs were performed using the Cytoscape plugin Molecular Complex Detection (MCODE) [18]. To perform the cluster analysis, the following parameters were used: node score cut-off = 0.2, degree cut-off = 2, k-score = 2, and max. depth = 100. KEGG pathway analyses were performed for both up- and downregulated genes using the online tool DAVID, with FDR-corrected *p* < 0.05 considered significant.

### 2.7. Cell Proliferation of THP-1 Cells Transfected with pcDNA-STX17-DT Recombinant Plasmid

The CCK-8 assay was employed to systematically monitor and compare the proliferative capacity of THP-1 cells over time after transfection with either the pcDNA-STX17-DT recombinant plasmid or the corresponding empty vector. Cell preparation and transfection procedure were the same as described above. Following transfection, cells were first incubated for 24 h to permit robust expression from the plasmids. Subsequently, viability was assessed at 0, 24, 48 and 72 h post-transfection. At each time-point, 10 µL CCK-8 reagent (Dojindo Molecular Technologies, Kumamoto, Japan) was added per well and the plate was returned to the 5% CO_2_ incubator for 1 h. Absorbance at 450 nm was then recorded on a microplate reader. Each measurement was performed in triplicate.

### 2.8. Apoptosis in Transfected THP-1 Cells

TUNEL (terminal deoxynucleotidyl transferase dUTP nick-end labeling) staining was performed to quantify and compare the extent of apoptosis in transfected THP-1 cells. Transfected THP-1 cells (pcDNA-STX17-DT and empty-vector controls) were seeded at 500 µL per well onto coverslips positioned in triplicate wells of a 6-well plate and incubated for 5 h (37 °C, 5% CO_2_) to allow attachment. Five hours later, gently add 2 mL of RPMI-1640 supplemented with 10% FBS to each well without disturbing the cells. Maintain the culture for a further 24 h at 37 °C, 5% CO_2_. Thereafter, aspirate the medium and wash the cells twice with PBS, 5 min per wash. Fix the cells with 4% paraformaldehyde at room temperature for 30 min, then rinse once with PBS. Permeabilize the cells with PBS containing 0.3% Triton X-100 for 5 min at room temperature, followed by two 5 min washes with PBS. Overlay each coverslip with the TUNEL reaction mix (TdT + fluor-conjugated dUTP; Beyotime, Shanghai, China) and incubate for 60 min at 37 °C in a humidified, light-tight chamber. After incubation, gently blot off the TUNEL reaction mixture with absorbent paper. Immediately apply DAPI-containing antifade mounting medium onto the coverslips and incubate at room temperature for 5 min to complete nuclear staining. Finally, mount the coverslips onto glass slides with the same medium and seal for imaging. Examine the slides under a fluorescence microscope (Olympus, Evident Corporation, Tokyo, Japan) using standard DAPI and green-fluorescence filter sets. Capture non-overlapping fields at 20× or 40× objective. In each field, count the total number of DAPI-stained nuclei and the number of TUNEL-positive nuclei. Evaluate a minimum of five randomly selected fields per coverslip. Calculate the percentage of apoptotic cells using the following formula: apoptotic rate (%) = (number of TUNEL-positive cells/total number of cells) × 100.

## 3. Statistical Analysis

All experiments were conducted with a minimum three replicates. The experimental data were analyzed with SPSS 19.0 software. Data were presented as mean ± standard deviation (Mean ± SD). Comparisons between two groups were performed with an unpaired two-tailed Student’s *t*-test, while comparisons among multiple groups, one-way analysis of variance (one-way ANOVA) was applied. Results were considered statistically significant at *p*-value < 0.05. The statistical charts were generated with GraphPad Prism 7.

## 4. Results

### 4.1. STX17-DT Expression in Transfected THP-1 Cells

**Figure 1:**
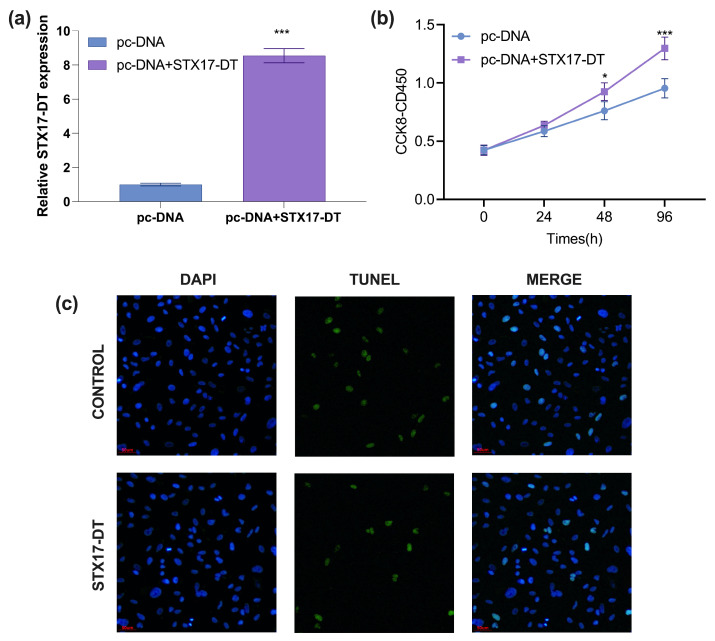
(**a**) RT-qPCR analysis of transfection efficiency of STX17-DT in THP-1 cells (mean ± SD, *** *p* < 0.001). (**b**) The THP-1 cell proliferation treated with STX17-DT overexpression. (mean ± SD, *** *p* < 0.001. (**c**) The TUNEL staining (green color indicative of necrosis/apoptosis; magnification × 400) and PI staining (blue color indicating nucleus deposition; magnification × 400) of THP-1 cells from STX17-DT or control group.

After transfection with pcDNA-STX17-DT for 48 h, RT-qPCR analysis of transfection efficiency showed that STX17-DT expression level in THP-1 cells was significantly increased, with an approximately 8-fold change compared to the pc-DNA3.1 empty vector group (mean ± SD, *p* < 0.00, Figure 1a), confirming that the recombinant plasmid was suitable for functional overexpression studies.

### 4.2. Cell Proliferation and Apoptosis of THP-1 Cells After Transfected with pcDNA-STX17-DT

To assess the impact of STX17-DT overexpression on cell proliferation, THP-1 cells were transfected and monitored by CCK-8 assay for 24–96 h. The absorbance (OD450 nm) was measured after CCK-8 incubation. The THP-1 cells treated with STX17-DT overexpression significantly enhanced viability at 48 h (1.22-fold; *p* < 0.05) and 96 h (1.36-fold; *p* < 0.001) relative to the control, demonstrating a time-dependent proliferative effect (mean ± SD, *** *p* < 0.001, Figure 1b).

To determine whether STX17-DT modulates apoptosis, THP-1 cells were transfected with STX17-DT overexpression plasmid for 48 h and analyzed by TUNEL staining. Compared to the empty vector control group, STX17-DT overexpression markedly reduced the proportion of TUNEL-positive nuclei, indicating a robust anti-apoptotic effect (green color indicative of necrosis/apoptosis; blue color indicating nucleus deposition, magnification ×400, Figure 1c).

### 4.3. RNA-Seq Data Processing and Reference Genome Alignment

We performed reference-based RNA-seq on six samples, generating a total of 41.73 G of Clean Data. The effective data for each sample ranged from 6.9 to 7.02 G. The Q30 base percentage ranged from 95.86% to 96.39%, with an average GC content of 53.39%. By aligning the reads to the reference genome, we obtained the genome alignment results for each sample, with alignment rates ranging from 98.12% to 98.55%.

### 4.4. Differential Gene Expression Analysis Between Transfected and Control Groups

**Figure 2:**
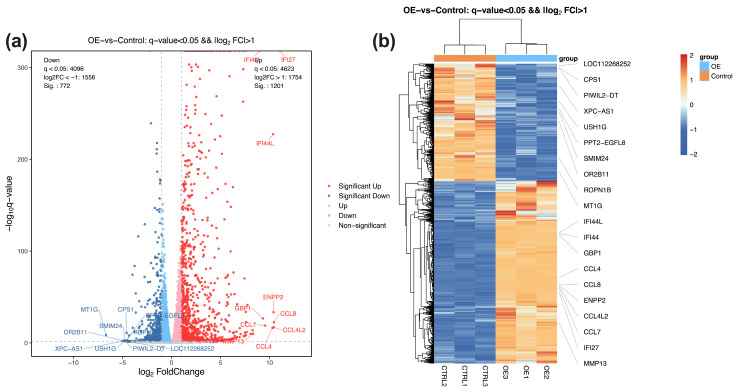
(**a**) The volcano map illustrates the RNA-seq analysis results between the transfected and control groups, including 1201 upregulated genes (red) and 772 downregulated genes (blue). Light red and blue dots denote non-significantly up-/down-regulated genes. (**b**) Heatmap analysis of the top ten upregulated and downregulated genes in the transfected OE and control groups. Orange denotes high relative expression; blue denotes low relative expression.

Following quality control, filtering, alignment, and normalization, a total of 1973 statistically significant differentially expressed genes (DEGs) were detected and, among which 1201 genes were upregulated and 772 genes were downregulated compared to the control group (q value < 0.05, |log_2_FC| > 1.0). The volcano plot in Figure 2a depicts the global distribution of DEGs, and the heat map in Figure 2b highlights the top 20 DEGs, including upregulated genes *IFI44L*, *IFI44*, *GBP1*, *CCL4*, and *CCL8*, and downregulated genes *LOC112268652*, *CPS1*, *PIWIL2-DT*, *XPC-AS1*, and *USH1G* in the STX17-DT transfected group.

### 4.5. Bioinformatic Function Enrichment Analysis of DEGs

**Figure 3:**
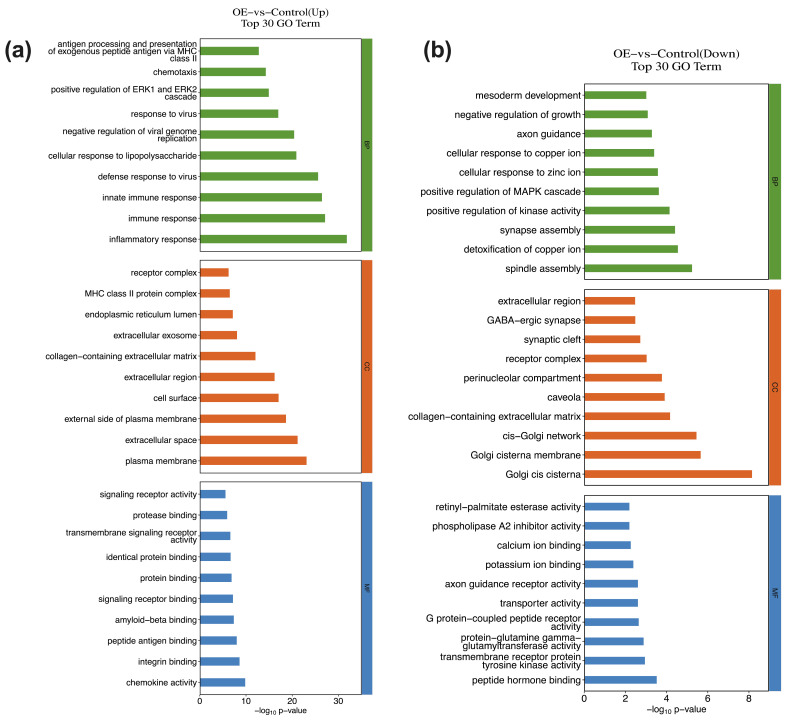
GO enrichment analysis of DEGs in THP-1 cells infected with STX17-DT versus control group ((**a**) upregulated; (**b**) downregulated). For each category (biological process, cellular component, molecular function), the top enriched terms are displayed, the horizontal axis represents the significance −log_10_ (*p*-value).

For each GO categories (biological process, cellular component, molecular function), terms with PopHits ≥ 5 were ranked by their corresponding −log_10_(*p*-value), and the top 10 terms were retained. Among upregulated genes, the most significantly enriched biological processes comprise inflammatory response, immune response, innate immune response, defense response to virus, and cellular response to lipopolysaccharide (Figure 3a). Downregulated genes were primarily involved in processes such as spindle assembly, detoxification of copper ion, synapse assembly, positive regulation of kinase activity, positive regulation of MAPK cascade (Figure 3b). At the molecular function level, upregulated genes were associated with chemokine activity, integrin binding, peptide antigen binding, amyloid-beta binding, and signaling receptor binding. In contrast, downregulated genes showed enrichment for peptide hormone binding, transmembrane receptor protein tyrosine kinase activity, protein-glutamine gamma glutamyltransferase activity, G-protein coupled peptide receptor activity, and transporter activity. For cellular component, the top annotations for the upregulated genes comprised the plasma membrane, extracellular space, external side of plasma membrane, cell surface, extracellular region. The downregulated genes were enriched in Golgi cis cistema, Golgi cistema membrane, cis-Golgi network, collagen-containing extracellular matrix, and caveola.

**Figure 4:**
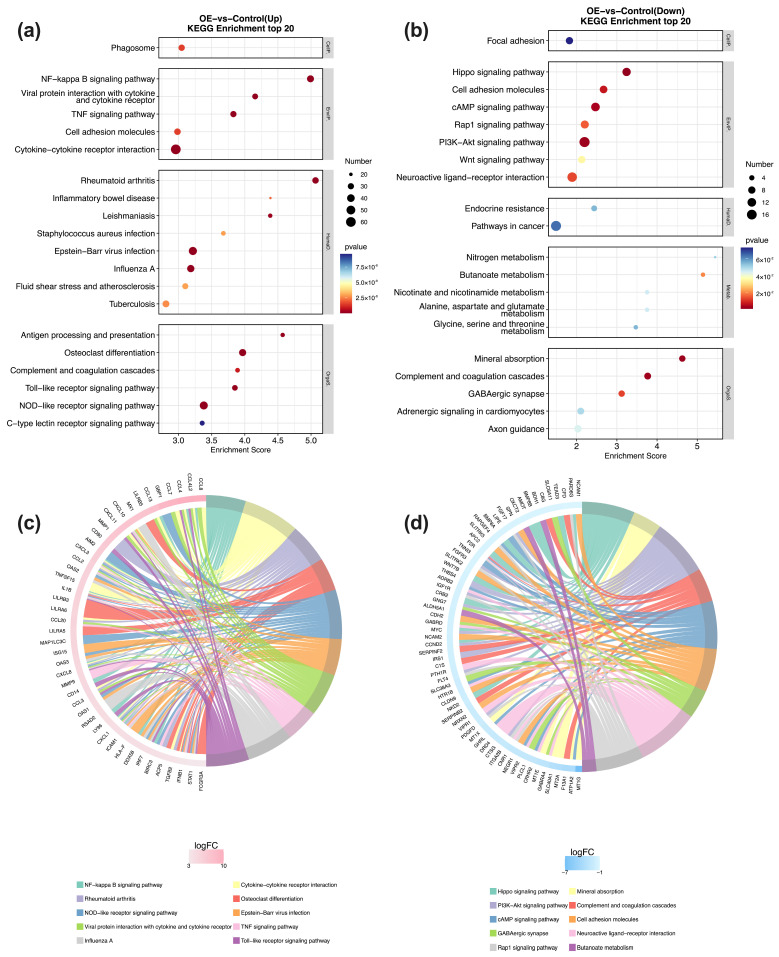
KEGG pathway enrichment analysis of DEGs. Bubble plots (**a**,**b**) show the most significantly enriched KEGG pathways for up- and downregulated genes, with bubble size representing gene count and color indicating the −log_10_ (*p*-value). Chord diagrams (**c**,**d**) depict the gene–pathway association network for the top 10 enriched terms, where ribbons link pathways to their constituent DEGs, and ribbon width is proportional to the number of connected genes.

KEGG pathway analysis (Entries with Pop Hits ≥ 5) of the DEGs revealed distinct signaling cascades altered by STX17-DT overexpression. Specifically, in the STX17-DT group, upregulated genes were significantly enriched in immune-inflammatory pathways, including NF-κB signaling, TNF signaling, Toll-like receptor signaling, and NOD-like receptor signaling. Enrichment in rheumatoid arthritis, cytokine–cytokine receptor interaction, osteoclast differentiation, and Epstein–Barr virus infection further underscores a pervasive pro-inflammatory state. Concurrently, the downregulated genes in the STX17-DT group pointed to a suppression of diverse homeostatic processes, such as PI3K-Akt signaling, cAMP signaling, complement and coagulation cascades, and metabolic pathways including mineral absorption and butanoate metabolism. The coordinated downregulation of Hippo and Rap1 signaling pathways, along with cell-adhesion molecules and neuroactive ligand–receptor interaction, suggests a broad impact on cell growth, adhesion, and communication. These contrasting profiles showed in Figure 4a–d, indicate that STX17-DT overexpression may contribute to an immunogenic transcriptome coupled with the repression of multiple regulatory and metabolic programs.

**Figure 5:**
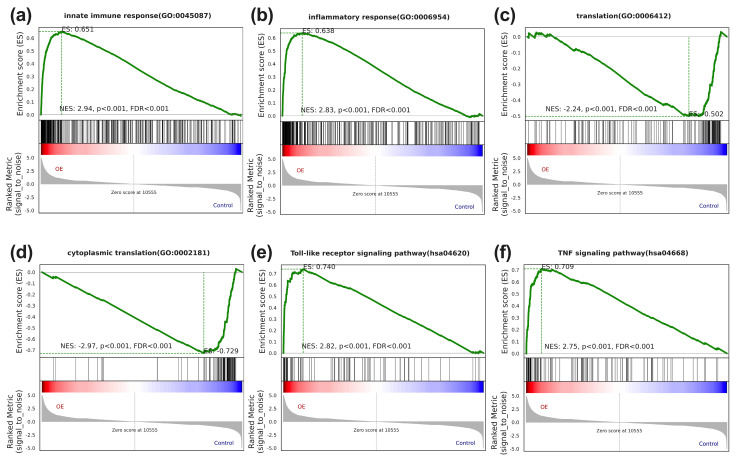
The gene set enrichment analysis of STX17-DT overexpression versus control THP-1 cells. GSEA plots demonstrate significant positive enrichment of the immune-related sets (innate immune response (**a**), inflammatory response (**b**), Toll-like receptor signaling pathway (**e**), and TNF signaling pathway (**f**) in the STX17-DT group), and negative enrichment of gene sets involved in translation-related sets (“translation” (**c**) and “cytoplasmic translation” (**d**)). In each plot, the green curve depicts the enrichment score (ES), black vertical lines mark member genes in the ranked gene list, and the bottom heatmap shows the expression level of the core genes. NES, normalized enrichment score; FDR, false discovery rate.

The GSEA suggested pronounced enrichment in innate immune and inflammation response gene sets within the upregulated core genes of STX-17 DT group (Figure 5a,b). In contrast, the down-regulated genes were dominated by translational processes (Figure 5c,d). Concordantly, GSEA kegg analysis further identified the Toll-like receptor and TNF-α signaling pathways as the top enriched pathways (Figure 5e,f).

### 4.6. TF Target Genes

**Figure 6:**
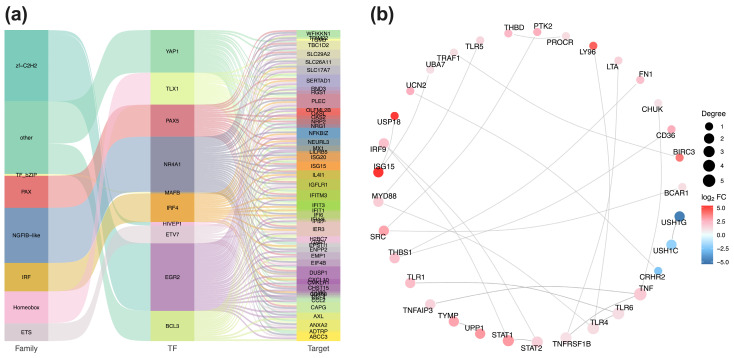
(**a**) TFs and target gene regulatory network. Sankey diagram illustrates the hierarchical relationships (TF families, individual TFs, target genes) among differentially expressed transcription factors (TFs) and their target. Connection width is proportional to the number of target genes (**b**) PPI network of key hubs. Protein–protein interaction network depicts the top 30 hub genes derived from the DEGs. Red and blue nodes represent up- and downregulated genes, respectively; node size corresponds to connectivity degree.

Based on established transcription factors and target genes relationships, we extracted the network of differentially expressed transcription factors (TFs) and their target DEGs. A Sankey diagram visualizes these interactions (Figure 6a). STX17-DT overexpression was associated with alterations in seven transcription factor families. The top 10 differentially expressed TFs included *YAP1*, *TLX1*, *PAX5*, *NR4A1*, *MAFB*, *IRF4*, *HIVEP1*, *ETV7*, *EGR2* and *BCL3*, and their predicted target DEGs, such as *SLC292A*, *SERTAD1*, *PLEC*, *BND3*, *NFKB1Z*, *ISG15*, *IL411*, *IFIT3* and *CAPG*, were predominantly enriched.

### 4.7. PPI Network Analysis

To identify core regulatory modules, PPI networks were constructed using the STRING database, based on curated species annotations or sequence homology (BLAST (version 2.16.0) e-value < 1 × 10^−10^). The most densely connected nodes among up- and downregulated DEGs were extracted, and the 30 interactions with the highest confidence scores were extracted for visualization (Figure 6b). Within it, the upregulated DEGs formed the distinct hub, centered on key immune mediators including *TNF*, *TLR6*, *TLR4*, *TNFRSF1B,* and *STAT2*. Conversely, *USH1G*, *USH1C,* and *CRHR2* constituted the core of the downregulated sub-network.

## 5. Discussion

This study, for the first time, provides evidence that overexpression of the long non-coding RNA STX17-DT in human monocytic cell line THP-1 orchestrates extensive transcriptomic reprogramming, with 1973 DEGs identified (1201 upregulated and 772 downregulated). The overwhelming enrichment of immune-related pathways among upregulated DEGs—particularly NF-kB signaling, Toll-like receptor cascades, and TNF signaling pathways strongly implicate STX17-DT may act as a critical modulator of innate immunity in PBC pathogenesis. Key upregulated genes such as *IFI44L*, *IFI44*, *GBP1* and chemokines *CCL4/CCL8*, all implicated in myeloid cell activation and recruitment. This pro-inflammatory molecular signature partially reflects an aspect of PBC histopathology, as multiple immune cells, such as regulatory T cells and mononuclear cells, infiltrate around bile ducts, which may contribute to the aberrant immune targeting of biliary epithelial cells. [9,19]. Notably, the significant enrichment of defense response to virus and cellular response to lipopolysaccharide suggests STX17-DT may trigger pattern-recognition receptors (e.g., *TLR4*), potentially via molecular mimicry of pathogen-associated molecular patterns [20,21,22]. This could offer new insights into the persistent, self-directed inflammation characteristic of PBC, even in the absence of overt infection [23,24,25]. The concordance between our in vitro findings and the in vivo PBC microenvironment underscores the relevance of the THP-1 model for initial mechanistic exploration [15].

Beyond transcriptomics, functional assays demonstrate that STX17-DT directly enhances monocyte/macrophage viability. CCK-8 assays revealed significantly increased proliferation in STX17-DT-overexpressing cells at 48 h and 96 h post-transfection. Crucially, TUNEL assays confirmed substantially reduced apoptosis, indicating that STX17-DT promotes both growth and survival. This phenotype correlates with DEG patterns: the downregulation of pro-apoptotic pathways (e.g., PI3K-Akt) and altered expression of proliferation-associated transcription factors like *EGR2* (early growth response 2) and *IRF4* (interferon regulatory factor 4) identified in the TF-target network. PPI network analysis further pinpointed highly interconnected hubs (*TNF*, *STAT2*, *TLR4*, *TLR6*), suggesting STX17-DT sustains inflammatory cell populations by potentiating NF-κB/STAT signaling pathways known to confer resistance to apoptosis in activated immune cells [26,27]. Collectively, these data propose a model wherein STX17-DT prolongs cell survival and amplifies the chronic inflammation of monocytes/macrophages infiltrating portal tracts, thereby exacerbating BEC injury and ductopenia. This mechanism offers a plausible explanation for the chronicity of portal inflammation observed in PBC patients, even those responding partially to UDCA [28].

The broad transcriptomic reprogramming induced by STX17-DT suggests it may act as a regulatory node, potentially influencing transcription factor activity; however, its exact molecular mechanism remains to be elucidated. Our analysis identified significant disturbances in TF families, with *YAP1*, *NR4A1*, and *MAFB* among the top altered regulators. Their predicted target DEGs (*ISG15*, *NFKB1Z*, *IL411*) are potent immune modulators. This scaffolding role reflects the function of other PBC-associated lncRNAs, such as H19, which drives macrophage activation and fibrosis [29]. Importantly, STX17-DT overexpression also suppressed metabolic pathways which are critical for immune homeostasis, including mineral absorption, complement and coagulation cascades, and cAMP signaling. The downregulation of butanoate metabolism (involving short-chain fatty acids, SCFAs) is particularly intriguing, as SCFAs are microbial metabolites known to suppress inflammation and promote regulatory T-cell function [30,31,32]. This bi-directional impact, enhancing inflammatory signals while suppressing immuno-regulatory and metabolic checkpoints, positions STX17-DT as a hub in PBC immunometabolic dysregulation. It may partially explain the variable therapeutic efficacy of UDCA, which primarily targets bile acid toxicity but may not fully counteract this profound immune reprogramming orchestrated by lncRNAs [33,34,35].

While this study provides possible evidence for the role of STX17-DT role in monocyte dysfunction, several limitations warrant careful interpretation. Firstly, though valuable for studying monocyte biology, THP-1 cells are a leukemic cell line. They may not fully replicate the behavior of primary human liver macrophages (Kupffer cells) or reflect the complex multicellular crosstalk within the liver microenvironment (e.g., hepatic stellate cells, cholangiocytes, or immune cells). Validating key findings in PBMCs from PBC patients and healthy controls, or in advanced co-culture systems (for example, THP-1 cells with primary human cholangiocytes), is essential. Secondly, while RNA-seq identified DEGs and pathways, the exact molecular mechanism by which STX17-DT exploits its effects remains elusive. Whether it acts as a competing endogenous RNA (ceRNA) sponging miRNAs, or directly interacts with chromatin modification complexes or transcription factors, needs to be clarified. Future work using techniques like RIP-seq or CRISPR-based methods is required to identify its direct binding partners and genomic targets. The observed transcriptomic changes may arise from the direct actions of STX17-DT or result from secondary effects of altered cell state like proliferation. Distinguishing primary targets requires kinetic studies and potentially single-cell RNA-seq after acute induction. Thirdly, the critical connection between our in vitro findings and disease development needs to be strengthened. Future work should correlate STX17-DT expression levels and activities with AMA status, disease stage, and treatment response, especially in UDCA non-responders. Lastly, generating transgenic mice or utilizing PBC mouse models with STX17-DT knock-down/overexpression, and validating these findings in PBC histological and plasma samples are crucial to conclusively establish causality and assess its influence on cholangitis, ductopenia, and fibrosis development.

In conclusion**,** this study provides preliminary in vitro evidence that the long non-coding RNA STX17-DT is a potential modulator of innate immune and inflammatory responses in monocytic THP-1 cells. Overexpression of STX17-DT promoted a pro-inflammatory transcriptomic signature, enhanced proliferation, and suppressed apoptosis in this cell model. These observations suggest that STX17-DT could contribute to sustaining inflammatory monocyte activity, a plausible mechanism that may be relevant to the persistent portal inflammation observed in PBC, which warrants further validation in more physiologically relevant systems.

## Data Availability

The raw data supporting the conclusions of this article will be made available by the corresponding author on request.
